# CisCross: A gene list enrichment analysis to predict upstream regulators in *Arabidopsis thaliana*

**DOI:** 10.3389/fpls.2022.942710

**Published:** 2022-08-18

**Authors:** Viktoriya V. Lavrekha, Victor G. Levitsky, Anton V. Tsukanov, Anton G. Bogomolov, Dmitry A. Grigorovich, Nadya Omelyanchuk, Elena V. Ubogoeva, Elena V. Zemlyanskaya, Victoria Mironova

**Affiliations:** ^1^Department of Systems Biology, Institute of Cytology and Genetics SB RAS, Novosibirsk, Russia; ^2^Department of Natural Sciences, Novosibirsk State University, Novosibirsk, Russia; ^3^Department of Cell Biology, Institute of Cytology and Genetics SB RAS, Novosibirsk, Russia; ^4^Service of Information Technologies, Institute of Cytology and Genetics SB RAS, Novosibirsk, Russia; ^5^Department of Plant Systems Physiology, RIBES, Radboud University, Nijmegen, Netherlands

**Keywords:** multi-omics data integration, DAP-seq, proximal promoters, RNA-seq, transcription factor binding profiles

## Abstract

Having DNA-binding profiles for a sufficient number of genome-encoded transcription factors (TFs) opens up the perspectives for systematic evaluation of the upstream regulators for the gene lists. Plant Cistrome database, a large collection of TF binding profiles detected using the DAP-seq method, made it possible for Arabidopsis. Here we re-processed raw DAP-seq data with MACS2, the most popular peak caller that leads among other ones according to quality metrics. In the benchmarking study, we confirmed that the improved collection of TF binding profiles supported a more precise gene list enrichment procedure, and resulted in a more relevant ranking of potential upstream regulators. Moreover, we consistently recovered the TF binding profiles that were missing in the previous collection of DAP-seq peak sets. We developed the CisCross web service (https://plamorph.sysbio.ru/ciscross/) that gives more flexibility in the analysis of potential upstream TF regulators for *Arabidopsis thaliana* genes.

## Introduction

Transcription factors (TFs) activate, repress, or fine-tune transcription of their targets. As TFs bind short genomic regions with specific target sequences, transcription factor binding sites (TFBS), the list of TF target genes can be predicted by localizing the TFBS in promoters. Inversely, the regulatory regions of coordinately expressed genes possess TFBS for the same TFs, so that potential upstream regulators can be detected *via* statistical enrichment analysis.

TFBSs are localized with greater or lesser precision *via* a number of experimental and computational methods. A variety of next-generation sequencing techniques have been applied in recent years to detect genome-wide TF binding profiles. For example, chromatin immunoprecipitation assay with sequencing (ChIP-seq) captures genomic regions bound by a DNA-associated protein in a sample for certain tissue, cell type, or treatment (Johnson et al., 2007); the DNA Affinity Purification and sequencing (DAP-seq) approach detects the DNA fragments from genomic DNA libraries that are bound by an *in vitro* expressed TF (O’Malley et al., 2016). DAP-seq highlights all genome loci which can be potentially bound by a TF, and it can be applied to hundreds of TFs.

Chromatin immunoprecipitation assay with sequencing and DAP-seq TF binding profiles consist of extended TF binding loci, also called peaks. One can use a peak set to assess an enrichment in the regulatory regions of the candidate genes and to test if the TF could be their common upstream regulator. Large collections of peaks for hundreds of TFs (O’Malley et al., 2016; Zheng et al., 2019; Kolmykov et al., 2021; Hammal et al., 2022) may allow predicting the upstream regulators systematically. Enrichr, a comprehensive gene list enrichment analysis web server (Kuleshov et al., 2016), predicts mammalian transcriptional regulators using hundreds of mammalian TF binding profiles from ENCODE (Davis et al., 2018). In the plant field, the large collection of DAP-seq profiles for 529 *Arabidopsis thaliana* TFs (O’Malley et al., 2016) is used for the enrichment analysis in web services TF DEACoN (Harkey et al., 2020) and EAT-UpTF (Shim and Seo, 2020).

The primary processing of raw data from ChIP-seq or DAP-seq experiments includes the peak calling step, a computational method used to identify areas in the genome that have been enriched with aligned reads. Peak caller GEM (Guo et al., 2012) was used to process raw data of the DAP-seq experiment for the Plant Cistrome database (O’Malley et al., 2016). The peak calling tool MACS2 (Zhang et al., 2008) has been the most commonly used peak caller (Nakato and Sakata, 2021) with over 6,400 citations as of Apr. 2022. A recent benchmark study of several peak callers including MACS2 and GEM by the multiple quality metrics confirmed that although these two tools outperformed other peak callers, the MACS2 tool more often than any other tool possessed the first rank in quality metrics (Kolmykov et al., 2019). The quality of a peak set significantly influences subsequent enrichment analysis, thus, a peak calling pipeline should minimize possible errors. A benchmarking study of simulated and real ChIP-seq data (Thomas et al., 2017) proved that (1) the methods using windows of different sizes to scan a genome for potential peaks were more powerful than ones that did not, and (2) methods using a Poisson test to rank the candidate peaks were more powerful than those using a Binomial test. To rank the candidate peaks, the peak calling tools GEM/MACS2 apply Binomial/Poisson tests, respectively (Zhang et al., 2008; Guo et al., 2012). GEM reports the genomic positions of peaks centers, and peaks are deduced as windows of a certain length (200 bp) around these positions. MACS2 uses the windows of multiple widths to scan a genome for candidate peaks and produces a set of peaks with carefully adjusted lengths. Apparently, MACS2-processed peak sets are more relevant for the gene list enrichment analysis than GEM-processed.

For Arabidopsis, the enrichment tools TF DEACoN (Harkey et al., 2020) and EAT-UpTF (Shim and Seo, 2020) used DAP-seq peak sets from the Plant Cistrome database (O’Malley et al., 2016). Here we re-processed DAP-seq raw data, getting a collection of peak sets of better quality and bigger size (peak sets for dozens of TFs were recovered). Finally, we developed the CisCross web service that utilized the updated peak sets profiles collections on Arabidopsis for the gene list enrichment analysis to predict upstream regulators. The CisCross web service implements the approach that we applied earlier (Shi et al., 2021; Bobrovskikh et al., 2022). Overall, the CisCross web service provides the opportunity for careful and flexible data analysis, which potentiates a deeper insight into the mechanisms of gene transcription regulation.

## Materials and methods

### Datasets

All 931 raw datasets from DAP-seq collection (O’Malley et al., 2016) were downloaded from the GEO database (GSE60143). The Plant Cistrome TF binding profiles collection (processed by the GEM in O’Malley et al., 2016) consisted of 568 peak sets for 387 TFs. We collected the benchmark compilation of 114 uniformly processed RNA-seq datasets from the EBI Expression Atlas (Moreno et al., 2022; see Supplementary Table 1),^1^ and five RNA-seq datasets on auxin treatments on arabidopsis seedlings/roots were taken from Freire-Rios et al. (2020).

### DAP-seq data pre-processing

All 931 raw datasets (GSE60143) were processed and aligned with snakePipes (v. 2.5.6) (Bhardwaj et al., 2019). Reads were mapped to the TAIR10 genome with the following parameters of snakePipes “DNA-mapping tair10 -i {input.dir} -o {output.dir} –dedup –mapq 3 –fastqc –trim.” For these options, bowtie2 (v. 2.3.5) was the default alignment tool (Langmead and Salzberg, 2012). Peak calling was done by MACS2 (v. 2.2.6) (Zhang et al., 2008) or GEM (v. 3.4) (Guo et al., 2012). In case of MACS2 we used snakePipes with the following parameters “ChIP-seq -d directory_with_data –peakCaller MACS2 tair10 TABLE.yaml –peakCallerOptions ‘–qvalue 0.05’”, “– singleEnd” or “–pariedEnd” were also used, depending on the type of data. TABLE.yaml was constructed as described in https://snakepipes.readthedocs.io/en/latest/content/workflows/ChIP-seq.html. For all samples, we used control SRR2926068 or SRR2926069 depending on the data type. In case of GEM we used the following command “java -jar gem.jar –expt sample.sam –ctrl control.sam –f SAM –g tair10.chrom.sizes –d Read_Distribution_default.txt –q 5 –sl.” We filtered out the peak sets with the fraction of reads in peaks (FRIP, Landt et al., 2012) less than 0.01.

We merged the replicas (if available) using the IDR (irreproducible discovery rate) tool (Li et al., 2011) with the following parameters “idr –samples $rep1 $rep2 –output-file $idr_out –soft-idr-threshold 0.01 -i 0.01.” To form the final peak set, we applied the following procedure. If an idr-processed set of peaks contained more than 2000 peaks, or its size at least twice exceeded one for an individual replica with the maximal number of peaks, then we chose an idr-processed set. In other cases, we chose an individual replica with the maximal FRIP value.

Finally, we took in further analysis sets containing at least 200 peaks. As a result, we have got 608/577 peak sets for 404/393 TFs for MACS2/GEM versions of the DAP-seq collection.

### Transcription factors families assignment

TFs were assigned to the gene names and the gene families based on the Plant Cistrome, PlantTFDB 3.0, and Araport databases (Pérez-Rodríguez et al., 2010; Pruneda-Paz et al., 2014; O’Malley et al., 2016; Pasha et al., 2020). In case of information inconsistency among the databases, or lack of information, we used other databases and focused studies (see Supplementary Table 2). As the most general units, we utilized the superfamilies according to Riechmann et al. (2000) and Zheng et al. (2016).

### CisCross algorithm

**FIGURE 1:**
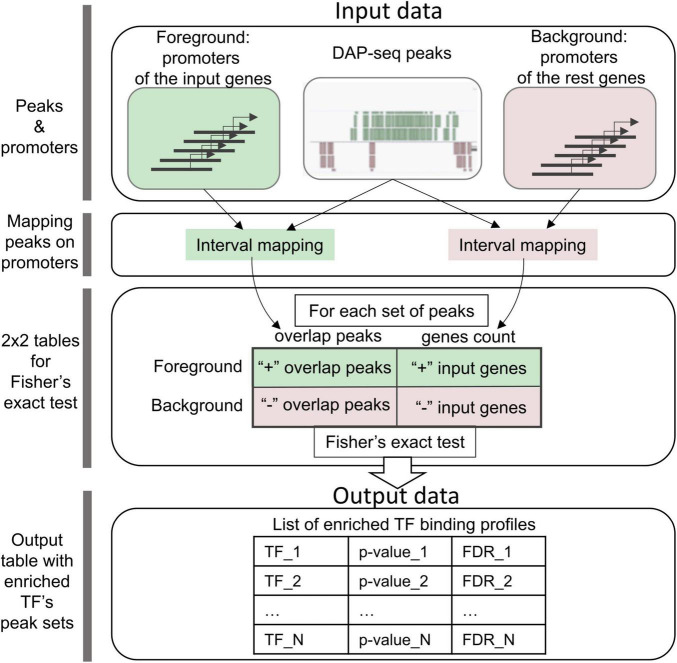
CisCross algorithm scheme (see section “Materials and methods”). Green/pink colors mark foreground and background data and respective parallel processes of their analysis. Foreground and background data comprise the annotations of promoter regions of the input genes and the rest genes, respectively. For one DAP-seq set of peaks, the first step of the analysis maps the peaks to promoters of the input genes and the rest genes. The second step uses these data of genome mapping to compile a 2 × 2 contingency table for the input genes and the rest genes with the counts of genes whose promoters overlap or do not overlap the peaks. Finally, Fisher’s exact test is applied to estimate the enrichment of the peaks in promoters (*p*-value). Output data comprise the list of enriched TF binding profiles in the ascending order of FDR (the significance *p*-value adjusted for multiple testing).

5′-regulatory regions of genes from an input list are used as the foreground and those for the rest of Arabidopsis genes as the background (Figure 1). For each TF peak set, CisCross counts the number of genes in the foreground/background, which 5′-regulatory regions overlap or do not overlap the TF binding peaks. The significance *p*-value of the input gene promoter enrichment for the peaks is assessed using Fisher’s exact test. These calculations are performed for each set of peaks. Finally, CisCross runs through all the peak sets in the selected DAP-seq collection and applies the correction for multiple testing to compute the False Discovery Rate (FDR) for each peak set.

### CisCross web service

The kernel of the CisCross web service is implemented in the Perl language. The user interface (input/output data) and running of the required Perl scripts were implemented in PHP language (version 7.4.3). The CisCross web service^2^ includes two modes: CisCross-Main and CisCross-Light. CisCross-Main implements the algorithm described above and on Figure 1 with the following options. For the background one can choose between Araport11 and TAIR10 annotations of the reference genome; whole-genome annotation of gene promoters as the regions of 500, 1000, 1500, 2000, or 2500 bp upstream to the transcription start sites. This annotation defines the foreground and background data as the promoters of the input genes and the rest genes. The available versions for the DAP-seq peak set collection are (1) GEM-processed Plant Cistrome (O’Malley et al., 2016); (2) GEM-processed (CisCross-GEM), this study; and (3) MACS2-processed (CisCross-MACS2), this study. Options for the multiple testing procedure are: Benjamini–Hochberg (Benjamini and Hochberg, 1995) or Bonferroni methods. Output data of CisCross-Main represent the list of potential upstream regulators in ascending order of the adjusted significance (FDR or adjusted *p*-value). CisCross-Light gives the list of DAP-Seq peaks detected in the 5′-regulatory region of the given input gene.

### Bed tracks treatment

We used the bedtools package (Quinlan and Hall, 2010) to estimate for a pair of peak sets (tracks) the fraction of the common overlapped length among the total genome length covered by any of them (the Jaccard statistics)^3^ and the significance of genomic co-localization of two tracks (evaluated by the *p*-value of Fisher’s exact test).^4^ We used Python’s package seaborn (v0.11.2) (Waskom, 2021) to visualize the distribution of the Jaccard statistic.

### *De novo* motifs search

We used the STREME tool (Bailey, 2021) for the *de novo* motifs search to confirm the quality of the peak sets from CisCross-GEM and CisCross-MACS2 versions of the DAP-seq collection. In *de novo* motif search we took DAP-seq datasets as the foreground datasets, and we compiled the background datasets from the randomly chosen sequences from the whole genome (Tsukanov et al., 2022).^5^ The significance of the similarity of enriched motifs to motifs of known TFs from the CIS-BP Arabidopsis motif collection (Weirauch et al., 2014) was proven using the motif comparison tool TOMTOM (*p*-value < 0.05; Gupta et al., 2007).

## Results

### Re-processing of DAP-seq TF binding profiles for *Arabidopsis thaliana*

**FIGURE 2:**
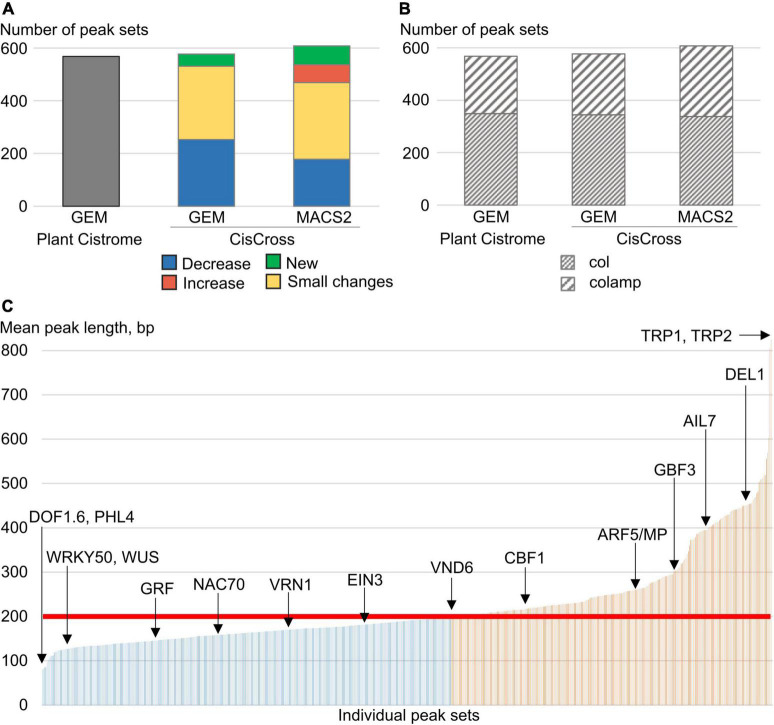
Summary statistics on the Plant Cistrome, CisCross-GEM, and CisCross-MACS2 versions of the DAP-seq peak set collection. **(A)** The total number of peak sets. “New” implies sets missing in the Plant Cistrome version. “Increase”/“Decrease” means that the number of peaks in sets increased/decreased at least twofold; “Small changes” indicates any smaller changes. *X*-axes in panels **(A,B)** denote the version of the DAP-seq collection. **(B)** The number of col and colamp peak sets in three versions of the DAP-seq collection. **(C)** Distribution of mean peak length (*Y*-axis) in individual peak sets (*X*-axis) for the CisCross-MACS2 version of the DAP-seq collection. Red line denotes the fixed peak length in the Plant Cistrome version (200 bp). Blue/orange colors mark the peak sets with shorter/longer mean peak length. A few example peak sets are named.

Firstly, we re-annotated raw DAP-seq data to make the Arabidopsis peak sets collection more relevant for the task of the gene list enrichment analysis. We used both GEM and MACS2 tools to pre-process raw DAP-seq data (see section “Materials and methods”). As a result, we’ve got three versions of the DAP-Seq peak sets collection processing: (1) 568 peak sets from Plant Cistrome (GEM, O’Malley et al., 2016) for 387 TFs; (2) new CisCross-GEM with 577 peak sets for 393 TFs; and (3) new CisCross-MACS2 with 608 peak sets for 404 TFs (Figure 2A). Note that re-annotation led to the recovery of the peak set for a dozen of TFs, e.g. CisCross-MACS2 includes 40 peak sets more than Plant Cistrome.

Plant Cistrome collection contained two types of peak sets that differed in the source genomic DNA libraries: (1) leaf gDNA possessing epigenetic DNA modifications (col data), and (2) leaf gDNA with methylcytosines eliminated due to PCR amplification (colamp data). Colamp data compared to col contains additionally about 180,000 peaks for TFBS spared from binding by DNA methylation (O’Malley et al., 2016). Compared to Plant Cistrome data, the MACS2-processing pipeline recovered novel TF binding profiles for 32 TFs in total (Supplementary Table 3 and Figure 2B). Among TFs with restored DNA-binding profiles, there are known important regulators such as WUSCHEL (WUS), ETHYLENE RESPONSE fACTOR 1 (ERF1), DEL1, and others (Table 1 and Supplementary Table 3). To test the relevance of generated peak sets we performed *de novo* motif search and compared the overrepresented motifs with the known TF binding sites (Supplementary Table 4). This procedure proved that all the peak sets are enriched in the binding sites for the respective TFs or their homologs.

**TABLE 1:** Changes in the number of peaks between selected TF binding profiles from the CisCross-MACS2 and Plant Cistrome versions of the DAP-seq peak set collection.

	TF name	TAIR ID	TF family	Number of peaks	
				Plant Cistrome	MACS2
**New**	DEL1	AT3G48160	E2F-DP	absent	382
	EIN3_colamp	AT3G20770	EIL	absent	774
	GATA1_colamp	AT3G24050	C2C2 (Zn)	absent	1926
	NAC16_colamp	AT1G34180	NAC	absent	1318
	WUS_colamp	AT2G17950	WOX	absent	2443
**Increase**	C3H67_colamp	AT5G63260	C3H (Zn)	4364	7551
	DAG2_colamp	AT2G46590	C2C2 (Zn)	9982	17182
	HB33_colamp	AT1G75240	ZF-HD	14246	25968
	OBP3_colamp	AT3G55370	C2C2 (Zn)	5038	20300
	SND2	AT4G28500	NAC	636	3695
**Small change**	FUS3	AT3G26790	B3-domain	3266	2055
	ERF19	AT1G22810	AP2/ERF	3765	4386
	LBD13_colamp	AT2G30340	LBD	3715	4437
	NAC62_colamp	AT3G49530	NAC	5883	5990
	RVE5	AT4G01280	MYB	9550	8793
**Decrease**	BBX31	AT3G21890	C2C2 (Zn)	16775	1654
	ATHB13	AT1G69780	HD-ZIP	23232	2173
	LBD23	AT3G26620	LBD	1451	383
	VND4	AT1G12260	NAC	10458	5058
	WRKY22	AT4G01250	WRKY	22769	7544

Next, we compared the Plant Cistrome, CisCross-GEM, and CisCross-MACS2 versions of the DAP-seq peak set collection. Supplementary Table 5 shows the distribution of the fraction of intersected regions and the significance of overlapping (the Jaccard statistics and *p*-value of Fisher’s exact test). We were unable to reproduce the Plant Cistrome data (O’Malley et al., 2016), as our version CisCross-GEM peak sets collection differed in size. Although Plant Cistrome had a greater total number of peaks (∼5.3 million peaks vs. ∼4.5 in CisCross-MACS2 and ∼3 in CisCross-GEM), it yielded the peak sets for a lesser number of TFs (Figure 2A). In CisCross-GEM, 30% of peak sets possessed a smaller number of peaks compared to those from Plant Cistrome. When comparing Plant Cistrome and CisCross-MACS2, the TF peak sets were classified into four groups: “New,” “Small changes,” “Decreased,” and “Increased” (Figure 2A and Table 1). There were 71 “New” peak sets. The “Increased” and “Decreased” groups contained the peak sets with more than twofold increase and decrease in the number of peaks, accordingly. The remaining peak sets were assigned to the “Small changes” group. The peak sets for most TF families were affected, however, there were some trends in how much reprocessing changed the peak set size. The peak sets for TCP, GARP, NAC, and SPL/SBP TF families did not change dramatically (“Small changes” group) while B3-domain, C2C2 (Zn), ZF-HD, and HD-ZIP TFs peak sets did (“Increased” or “Decreased” groups).

As it was expected, MACS2-generated peaks greatly vary in length (Figure 2C and Supplementary Figure 1). While the default peak length in GEM (200 bp) approximates well the average length of a peak (256 bp), some TFs show significantly longer peaks (TRP1-2, ARF5, AIL7), and some shorter ones (PHL4, WUS, WRKYs). The dependence between the mean peak length and the number of peaks for Plant Cistrome, CisCross-GEM, and CisCross-MACS2 versions of the DAP-seq peak collection proved that the fixed peak length of 200 bp used in GEM peak caller was a too rough estimate for a peak length (Supplementary Figure 2). The pairwise comparisons of the number of peaks in different DAP-seq TF binding collections (Supplementary Figure 2) proposed that a portion of MACS2-generated peaks experienced joining of neighboring GEM-processed peaks. E.g., longer peaks of MACS2 (>200 bp) may correspond to several tandemly arranged GEM peaks. The shorter MACS2 peaks (<200 bp) may correspond to GEM peaks of length 200 bp, whose flanking regions even do not contain reads, or several extra short MACS2 peaks may be joined in one GEM peak. Overall, the peak calling of MACS2 seems to be more careful compared to GEM in mapping the start/end of the peaks.

In the CisCross-GEM and CisCross-MACS2 versions of the DAP-seq collection, *de novo* motifs search (Bailey, 2021) resulted in the motifs with significant similarities (*p*-value < 0.05) to known matrices of respective target TFs or their homologs from the CIS-BP database (Weirauch et al., 2014; Supplementary Table 4). Since the overlap fractions between the CisCross-GEM/CisCross-MACS2 versions and the Plant Cistrome are moderate, though the fractions of genomics overlap are very significant for the overwhelming majority of the respective peak sets (Supplementary Table 5), it is reasonable to test all three versions of the DAP-seq collection in subsequent analysis. Though we expect that the CisCross-MACS2 collection should be more suitable for the gene list enrichment analysis since this collection provides a greater number of peak sets and the peaks lengths are detected more precisely.

### Benchmarking gene set enrichment analysis

**FIGURE 3:**
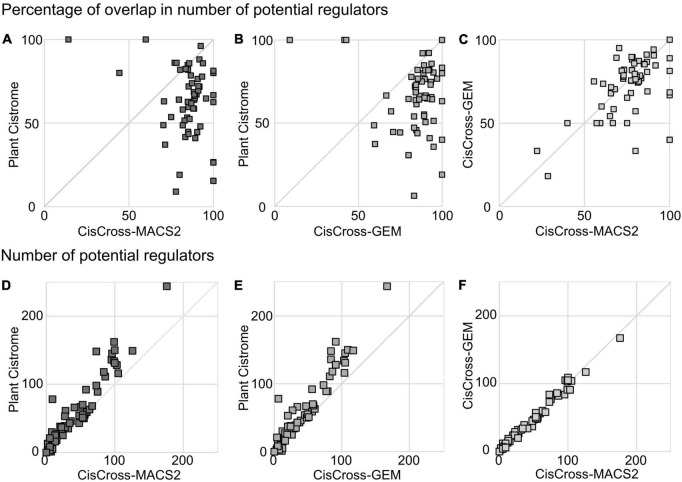
Comparison of the results for the gene list enrichment analysis in pairwise combinations of different versions of the DAP-seq collection for the benchmark compilation of RNA-seq data from the EBI Expression Atlas (see section “Materials and methods”). Panels **(A–C)** show the percentage of overlap of the output lists for potential TF regulators (FDR < 0.05). Panels **(D–F)** show the total number of potential TF regulators (FDR < 0.05).

Next, we performed the gene list enrichment analysis on the benchmark compilation of RNA-seq datasets for Arabidopsis from EBI Expression Atlas (Moreno et al., 2022). We used the CisCross algorithm for gene list enrichment analysis (Figure 1) and ran it with three versions of the DAP-seq peak set collection. The overlap between the lists of TF regulators generated for the same list of differentially expressed genes by different collection versions varied between 60 and 90% (Figures 3A–C). The pairwise comparisons of the number of potential TF regulators between Plant Cistrome, CisCross-GEM, and CisCross-MACS2 collection versions showed excellent positive correlations (Figures 3D–F). In particular, the pair “CisCross-GEM vs. CisCross-MACS2” respected the perfect diagonal linear trend, except for two outlier points (Figure 3F). In the two remaining pairs, the Plant Cistrome systematically predicted slightly more TF regulators than CisCross-GEM or CisCross-MACS2 (Figures 3D,E). These differences might come from the variation in the total number of TF regulators (568, 577, or 608, respectively, Figures 2A,B) that affected the FDR in the multiple testing correction, thus changing the cutoff threshold for the significant enrichment of an upstream regulator TF between the runs. Pairwise comparisons of the significance enrichment levels assessed for the same TFs with different versions of the DAP-seq peak sets collection (Supplementary Figure 3) confirmed that the differences in the number of potential regulators originated from low-scoring potential regulators defined with Plant Cistrome.

The fact that CisCross-MACS2 and CisCross-GEM collection versions had dozens of unique TF peak sets that were absent in Plant Cistrome (Figure 2A) also influenced the output. On the one hand, it made the multiple testing correction more stringent, as more independent tests run at the same time. On the other hand, new TF regulators whose peak sets were unavailable in the Plant Cistrome were found in 90% of the outputs for CisCross-MACS2. One example is the upregulated genes in *rhd6* mutant with defective development of the root hairs (Huang et al., 2017) among other potential TF regulators CisCross-MACS2 detected EIN3 (see Supplementary Table 6). This is a relevant result because EIN3 and RHD6 were shown to coordinatively regulate the root hair growth (Feng et al., 2017).

We also noted that the ranking of TFs upstream regulators by the significance of enrichment differed in the output data respecting three collection versions (Supplementary Table 6). We exemplify this in the analysis of the transcriptomic datasets for various treatment times of auxin hormone (Freire-Rios et al., 2020). With CisCross we tested the lists of auxin-activated genes for enrichment with TF binding profiles (see Supplementary Table 6). Table 2 shows the ranks for two TFs in the output: auxin regulator ARF5 and ethylene regulator EIN3. All three versions of the DAP-seq collection showed that ARF5 targets were the most overrepresented in the gene regulatory regions activated at the earliest time point (1 h of auxin treatment). The significant enrichment was observed for all treatment time points till the latest one (55 h); however, the rank of ARF5 was much lower in the late response suggesting that other TFs played major roles. Among these TFs, EIN3 appeared to be playing an important role in the late auxin response. CisCross-MACS2 detected EIN3 gradually improving its rank starting from 2 h of auxin treatment till the first rank at 55 h of treatment. Although all three DAP-seq collection versions showed concordant results for ARF5 and EIN3, CisCross-MACS2 performed the best in terms of the ranking relevance (Table 2).

**TABLE 2:** Gene list enrichment analysis of the series of transcriptomic data for various treatment times by auxin hormone (see section “Materials and methods”).

	1 h	2 h	4 h	6 h	55 h
**ARF5/MP**					
Plant Cistrome	1***	78***	23***	51	124**
CisCross-GEM	1***	42***	6***	74	114*
CisCross-MACS2	1***	81**	3***	54	40***
**EIN3**					
Plant Cistrome	68	145*	81*	81	2***
CisCross-GEM	340	168	179	7***	21***
CisCross-MACS2	37	115*	42*	2***	1***

The ranks for two TF regulators ARF5, and EIN3 are shown. The asterisk marks the significance of enrichment corrected with Benjamini-Hochberg (FDR), *** is FDR < 0.001, **FDR < 0.01, * < FDR < 0.1. The hits with the lowest rank for the TF over three versions of the DAP-seq collection are marked in bold.

### CisCross web service: Application modes and functionality

**FIGURE 4:**
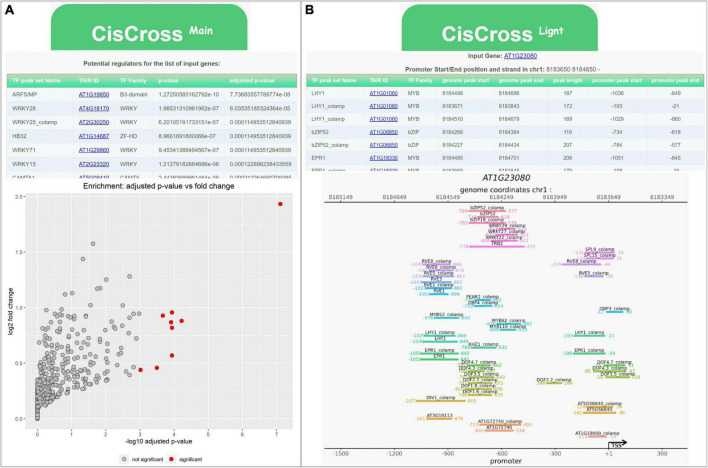
Examples of the output data for the CisCross web service. **(A)** CisCross-Main mode for gene list enrichment analysis (for the list of auxin up regulated genes from GSE149410). **(B)** CisCross-Light mode for the upstream region of *PIN7* (AT1G23080) gene.

As shown above, all three versions of the DAP-seq collection performed well in a gene list enrichment analysis; however, the list of potential TFs regulators and their ranking might significantly change in specific datasets. One should have the possibility to compare the output produced by three versions of the DAP-seq collections. We developed the CisCross web service (see text footnote 2) to solve this problem and to be more flexible in the gene list enrichment analysis for Arabidopsis. The CisCross functionality includes two application modes: CisCross-Main and CisCross-Light (Figure 4).

CisCross-Main applies the gene list enrichment procedure described above (Figure 1). To predict potential TF regulators, a list of genes is supplied as an input. The user should define several parameters, including a promoter length to build the foreground, the version of the DAP-seq collection, the release of genome annotation, and the type of multiple testing procedure, or use the default parameters values. The output data provide a list of potential TF regulators (Figure 1), sorted in ascending order of their significance. Other outputs include the gene-to-TF table and the scatterplot visualizing prediction results (Figure 4A).

CisCross-Light gives an overview of the TF binding regions detected in the upstream regions of an input gene. A user should define the promoter length and the version of the DAP-seq collection. This analysis suggests the list of potential upstream regulators with the genomic coordinates of their peaks detected by the DAP-seq experiment. Location of the DAP-seq peaks is presented in a table and as a genome map (Figure 4B).

## Discussion

Modern transcriptome sequencing technologies (RNA-seq, scRNA-seq) routinely generate thousands of lists of differentially expressed genes or marker genes. To better understand the transcriptomic changes one should have an access to user-friendly and flexible tools to search for the enriched features in these lists of genes. One of the standard procedures widely used in genomics is the Gene Ontology (GO) enrichment analysis. In the plant field, there are special web services to solve this task: AgriGO (Du et al., 2010), ShinyGo (Ge et al., 2020), or PANTHER (Mi et al., 2021). However, the enrichment analysis can be equally applied to other gene annotations, predicted features, and genome-scale experimental data. TF binding profiles resolved for hundreds of TFs by the DAP-seq experiment (O’Malley et al., 2016) is an appealing collection of genome-scale data that can be used to find the potential upstream regulators for a list of candidate genes. TF-Decon shiny app tool (Harkey et al., 2020) performs the gene list enrichment analysis for the Plant Cistrome dataset for TF targets. Although it is user-friendly and available online, one can not specify the promoter length or change the statistical analysis procedure there. An alternative command-line tool EAT-UpTF also utilizes the Plant Cistrome TF binding profiles (Shim and Seo, 2020). Although it is not available online and requires some programming skills, one can adjust some steps in the analysis, e.g., modify the length of the 5′-regulatory regions in the analysis. Both tools adopted the originally processed peaks of the Plant Cistrome collections which were prepared with the peak calling tool GEM (Guo et al., 2012).

Previously, we also used the Plant Cistrome peaks in our studies (Shi et al., 2021) and noted that their processing procedure was not quite correct. Namely, the authors pre-processed raw data with not the most popular peak calling tool GEM (Guo et al., 2012) that reported exact genomic positions of TF binding, and TF binding peaks were deduced as windows of a certain length (200 bp) around these positions. In a benchmark study (Thomas et al., 2017) MACS2 peak caller outperformed GEM in terms of sensitivity and precision. The MACS2 is the most popular and the sole peak caller that has been applied in all basic whole-genome TF binding profile annotation databases, e.g., ReMap (Hammal et al., 2022), CISTROME DB (Zheng et al., 2019), ChIP-Atlas (Zou et al., 2022), and GTRD (Kolmykov et al., 2021). What is important for the gene list enrichment analysis, MACS2 uses the windows of multiple widths to scan a genome for candidate peaks and produces a set of peaks with carefully adjusted lengths. Indeed, we observed major changes in the number of peaks and their length between the CisCross-MACS2 and the Plant Cistrome (Figure 2). Both software tools MACS2 and GEM were recommended to call peaks in the standard protocol of DAP-seq data processing (Bartlett et al., 2017).^6^ Some recent studies (da Silveira Falavigna et al., 2021; Lai et al., 2021a,b) applied the MACS2 tool for DAP-seq data processing for several TFs in Arabidopsis. The benchmark DAP-seq collection (O’Malley et al., 2016) was also re-processed with MACS2 in the ReMap and ChIP-Hub databases (Fu et al., 2022; Hammal et al., 2022).

Although the lists of upstream regulators detected using different versions of the DAP-seq collection were largely overlapped, we saw the diversity in the content and ranking of potential upstream regulators (Figure 3 and Table 2). We noted that some relevant regulators were detected only by CisCross-MACS2 or CisCross-GEM (e.g., the example with EIN3 detection for the activated genes in *rhd6* mutant, see Supplementary Table 6). And we noted the ranking of TF regulators being more relevant in CisCross-MACS2-processed output (Table 2). Anyway, as Plant Cistrome gave more hits for some gene lists, we cannot exclude that they might be relevant and someone would like to use these predictions. This is why we developed the CisCross web service that gives a user an opportunity to perform the gene list enrichment analysis with different settings, including the possibility to set the version of peak sets collection. In the future, we plan to upgrade it with the ChIP-seq data for Arabidopsis TFs.

## Data availability statement

Publicly available datasets were analyzed in this study. This data can be found here: https://www.ncbi.nlm.nih.gov/geo/query/acc.cgi?acc=GSE60143. Re-processed DAP-seq datasets, their overrepresented motifs, and the data on the TFs family assignment are available here: https://plamorph.sysbio.ru/ciscross/.

## Author contributions

VM and VGL designed the algorithm and supervised the project. AVT processed raw DAP-seq data and performed analysis of motifs. AGB, VVL, VM, DAG, and VGL developed the web service. VVL implemented the algorithm and performed the analysis of RNA-seq data. NO and EVU elaborated on the web service content. VVL, VM, and VGL wrote the manuscript. VVL, VM, EVZ, and VGL revised and finalized the manuscript. All authors contributed to manuscript completion and revision.

## Footnotes

1
https://www.ebi.ac.uk/gxa/home

2
https://plamorph.sysbio.ru/ciscross/

3
https://bedtools.readthedocs.io/en/latest/content/tools/jaccard.html

4
https://bedtools.readthedocs.io/en/latest/content/tools/fisher.html

5
https://github.com/parthian-sterlet/sitega

6
https://github.com/ndu-invitae/Dap-Seq-pipeline
