# Supplementary material for: CisCross: A gene list enrichment analysis to predict upstream regulators in Arabidopsis thaliana
Source: Front Plant Sci. 2022 Aug 18;13:942710. doi: 10.3389/fpls.2022.942710 (PMC9434332; doi:10.3389/fpls.2022.942710)
Supplement: Supplementary file 2 [file Image_1.pdf]

10.3389/fpls.2022.942710

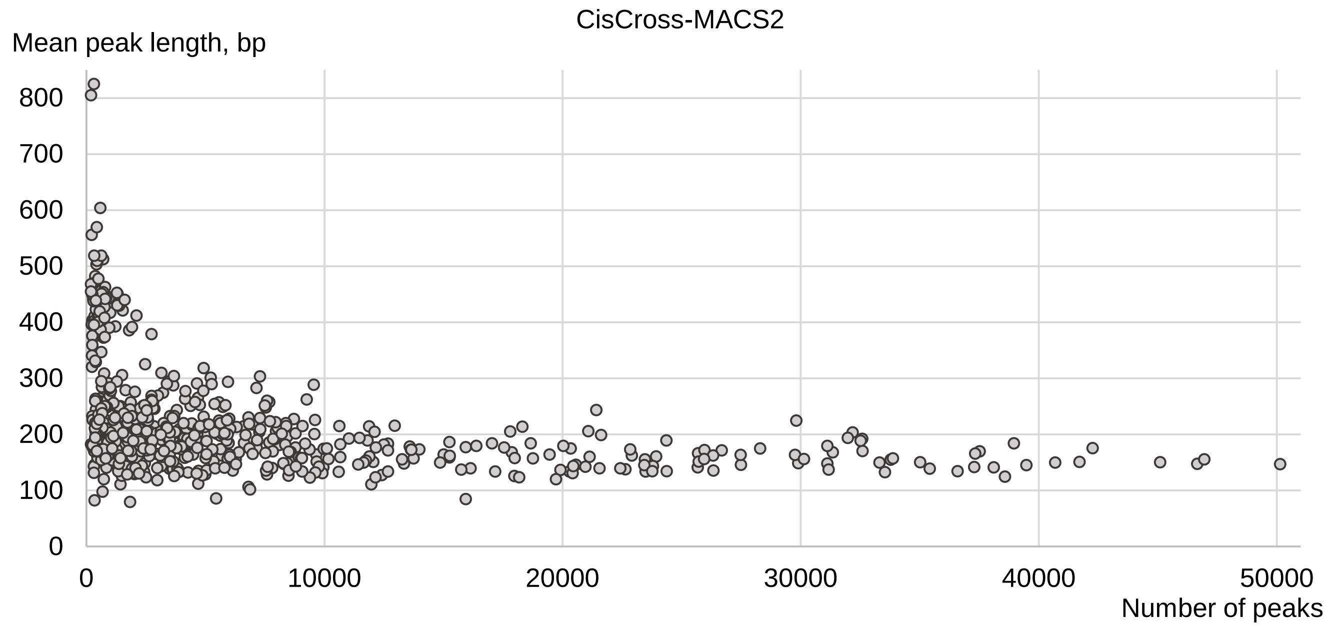

Figure S1. An average length of the peak (Y axis) relay to the number of peaks (X axis) in the individual TF peak set from the CisCross-MACS2 collection.
