# Supplementary material for: CisCross: A gene list enrichment analysis to predict upstream regulators in Arabidopsis thaliana
Source: Front Plant Sci. 2022 Aug 18;13:942710. doi: 10.3389/fpls.2022.942710 (PMC9434332; doi:10.3389/fpls.2022.942710)
Supplement: Supplementary file 3 [file Image_2.pdf]

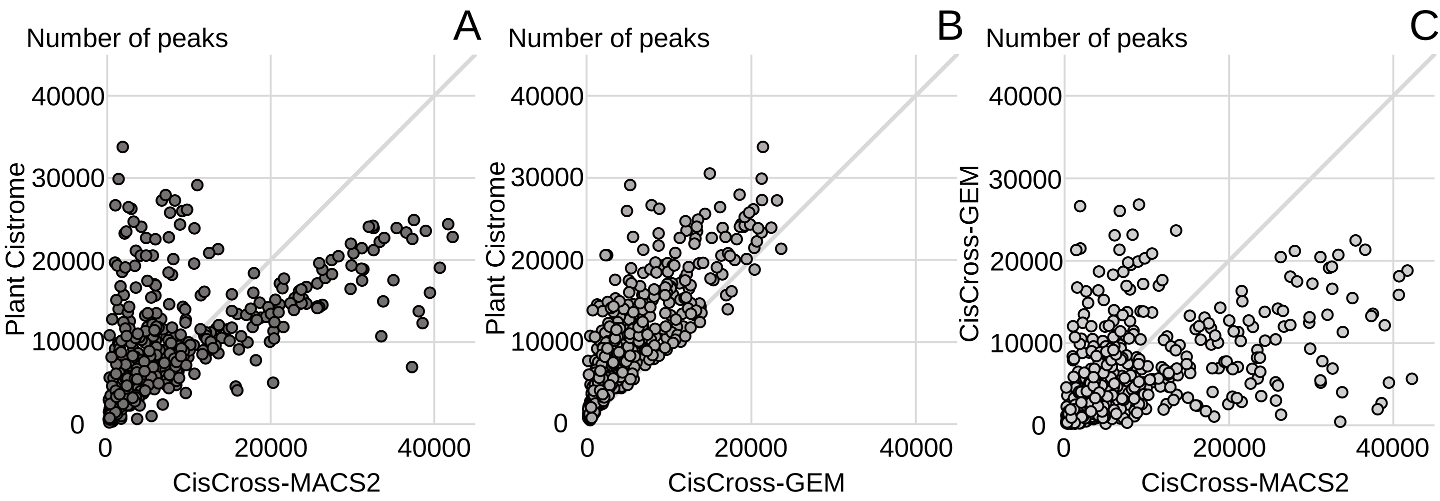

Figure S2. The pairwise comparisons of the number of peaks in different versions of the DAP-seq peak set collection. The number of peaks for each TF regulator and each version of the collection the CisCross algorithm computed by the multiple testing procedure (see Materials and Methods). Headers of X and Y axes imply the collection versions.
