# Supplementary material for: CisCross: A gene list enrichment analysis to predict upstream regulators in Arabidopsis thaliana
Source: Front Plant Sci. 2022 Aug 18;13:942710. doi: 10.3389/fpls.2022.942710 (PMC9434332; doi:10.3389/fpls.2022.942710)
Supplement: Supplementary file 4 [file Image_3.pdf]

 10.3389/fpls.2022.942710

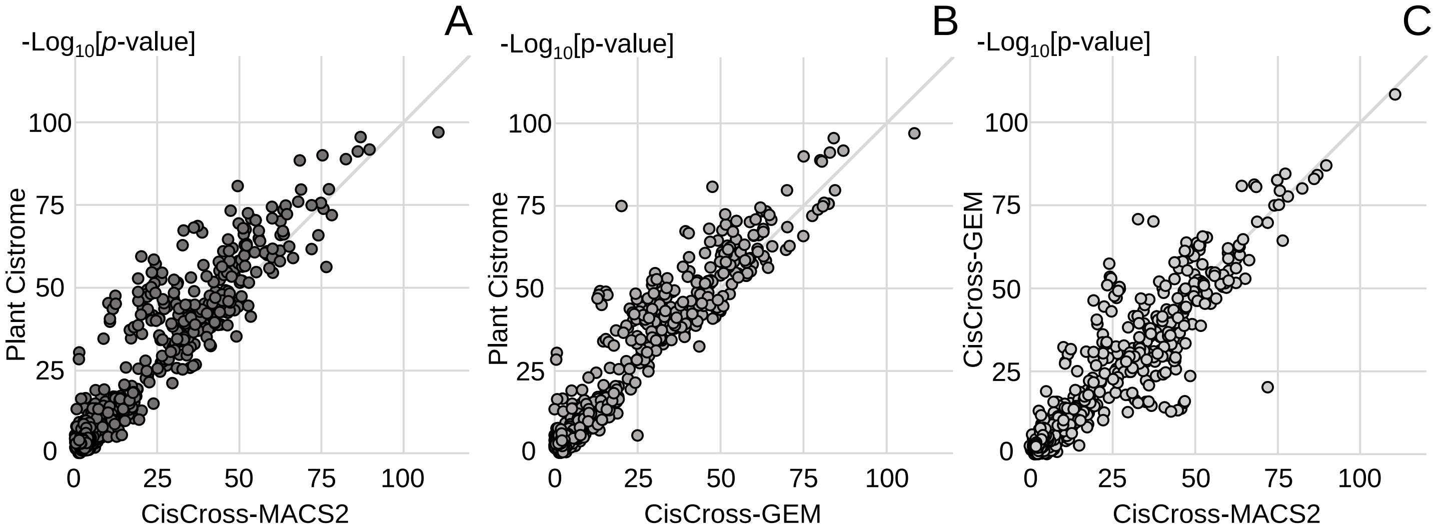

Figure S3. Comparison of the results for the gene list enrichment analysis in pairwise combinations of different versions of the DAP-seq collection for the benchmark compilation of RNA-seq data from the EBI Expression Atlas (see Materials and Methods). X and Y axes in all plots show the significance of TF regulators (-Log10[*p*-value]) for different versions of the DAP-seq collection (*p*-value<0.05, see Materials and Methods).
