# Supplementary material for: CisCross: A gene list enrichment analysis to predict upstream regulators in Arabidopsis thaliana
Source: Front Plant Sci. 2022 Aug 18;13:942710. doi: 10.3389/fpls.2022.942710 (PMC9434332; doi:10.3389/fpls.2022.942710)
Supplement: Supplementary file 5 [file Image_4.pdf]

10.3389/fpls.2022.942710

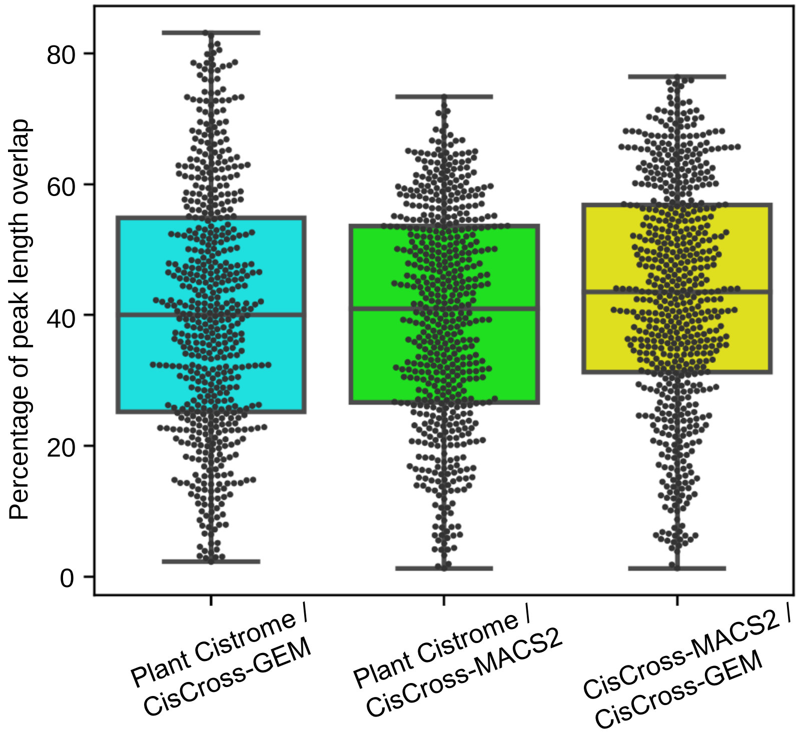

Figure S4. Distributions of the fractions of the overlapped peak length for the pairwise comparisons among three versions of the DAP-seq peak collection (the Jaccard statistics, see Materials and Methods). The Y axis shows the percentage of peak length overlap.
